# Non-Surgical Root Canal Treatment of Dens Invaginatus 3 in a Maxillary Lateral Incisor

**Published:** 2008-04-02

**Authors:** Saeed Moradi, Zakyeh Donyavi, Mohammad Esmaealzade

**Affiliations:** 1 *Department of Endodontics, Dental School, Mashad University of Medical Sciences, Mashad, Iran*; 2 *Department of Pediatric Dentistry, Dental School, Mashad University of Medical Sciences, Mashad, Iran*

**Keywords:** Dens Invaginatus, Lateral Incisor, Maxillary, Root Canal Treatment

## Abstract

The aim of this case report was to describe the clinical management of an unusual dens invaginatus type 3. A case of dens invaginatus in a maxillary lateral incisor with a periapical lesion is reported. The patient presented with pain and localized swelling. Despite the complex anatomy and diagnosis of dens invaginatus, non-surgical root canal treatment was performed successfully.

**Key Learning Points:**

- Dens invaginatus may be presented in different forms, and the etiology of this phenomenon is not fully understood.

- Due to abnormal anatomical configuration, dens invaginatus presents technical difficulties in its clinical management.

- Non-surgical root canal treatment can be performed successfully.

## INTRODUCTION

Dens invaginatus also called dens in dente, dilated composed odontoma or gestant odontoma, is a developmental disturbance resulting from invagination of the enamel organ toward the dental papilla before mineralization; it may be limited to the tooth crown or invade the root to affect the periapical region (1).

According to Pindborg, the etiology of this malformation is unknown; yet the following explanations have been proposed: (i) delayed focal growth, (ii) stimulation in the area of the tooth bud and (iii) abnormal pressure on tissues surrounding the dental organ (2).

The incidence ranges from 0.04% to 10% and primarily affects the permanent dentition, especially the maxillary lateral incisors, although primary teeth may also be affected (3,4).

Clinically, the crown of an affected tooth may appear normal or have size and shape alterations. Thus, there may be greater buccolingual diameter, peg-shaped or barrel-shaped teeth or a talon cusp. Mild invaginations exhibit only a lingual pit, which is often clinically unnoticed. However, when the crown is large and has a prominent cusp with a palatogingival groove, the occurrence of dens invaginatus is assumed (5-8).

**Figure 1:**
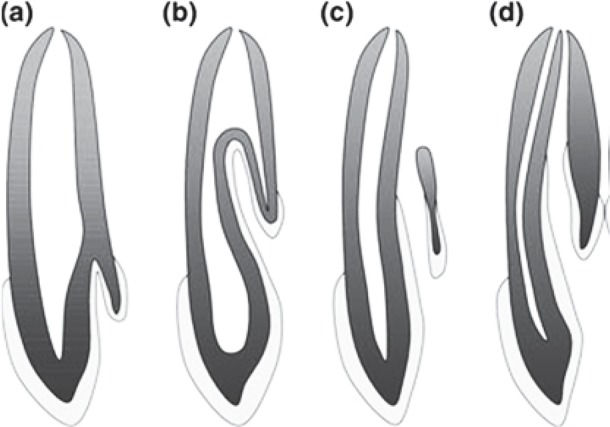
Classification of dens invaginatus. Adapted from Oehlers (1957) class I (a); class II (b) class III (c and d)

According to the extent of the invagination into the tooth structure, Oehlers proposed the following classification: type I, characterized by a small invagination limited to the crown not extending beyond the cementoenamel junction; type II, the line delineating enamel invagination invades the root, yet is limited to it as a ‘cul-de- sac’ configuration, without reaching the periodontal ligament, yet it may communicate with the tooth pulp and type III, a severe form of invagination extending through the root and ending at the apical region without direct communication with the tooth pulp(9) (Figure 1).

According to Bhatt and Dholakia, this root variability of dens invaginatus is usually related to folding of the Hertwig’s epithelial sheath (10). Radiographically, the roots present smaller dimensions with presence of a radiopaque formation with density similar to that of enamel which is invaginated from the cusp through variable extents into the root. This invagination varies in shape and size, and may present a loop-like or pear- shaped configuration or a slightly radiolucent structure, to more extensive and bizarre shapes, simulating a ‘tooth within a tooth’ (11-12). Hygiene around the area of invagination is difficult or impossible, which reveals the importance of early diagnosis, which may be completed from radiographs even before tooth eruption (13).

Histologically, the structure of dens invaginatus is composed of internal enamel, dentine, connective tissue nucleus and blood supply. The internal enamel is remarkably hypo mineralized and the dentine is uniformly mineralized highlighted that the thin canals or fissures connecting the invagination to the pulp cavity act as pathways through which microorganisms and irritating agents from the oral cavity may lead to pulp alterations before the development of caries (14).

An early diagnosis of such malformations is crucial.Due to abnormal anatomical configuration, an invaginated tooth presents technical difficulties in its clinical management. Many treatment regimens have been suggested such as conventional root canal treatment (3), combined root canal–surgical treatment (15), intentional replantation and extraction (16). However, extraction and intentional replantation are usually the last resort. Surgical approaches are avoided when possible, due to the inability to completely clean the entire canal and issues of patient comfort. Additionally, surgical intervention may pose the challenge of producing an apical seal in a thin, fragile and irregular apical root end.

The delicate root canal walls of the root-end preparation may fracture during compaction of root-end materials (17).

When no communication exists between the invagination and the pulp cavity, nonsurgical endodontic treatment has proved successfully by treating the invagination as a separate entity and preserving the vitality of the pulp. Several case reports have described successful endodontic treatment of periapical lesions in maxillary incisors with dens invaginatus type 3 (18,19).

The purpose of the present article is to describe a case of apical periodontitis associated with a tooth containing a dens invaginatus healed successfully after non-surgical root canal treatment.

## CASE REPORT

A 15-year-old girl was referred by her general dental practitioner (because of pain and swelling) to the Endodontic clinic of Mashad University, Iran, for evaluation and treatment of maxillary right lateral incisor. She reported throbbing pain and swelling from a week before, but at the time of examination, there were no symptoms.

Clinical examination revealed the maxillary lateral incisor to be unusually greater buccolingual diameter. Preoperative palatal inspection of maxillary lateral incisor confirmed the large enamel projection. There was no evidence of swelling or sinus tract; however the tooth was slightly tender to percussion.

The tooth was not responsive to CO_2_ stimulation whilst adjacent teeth respond normally. Periodontal probing was within normal limit.

**Figure 2:**
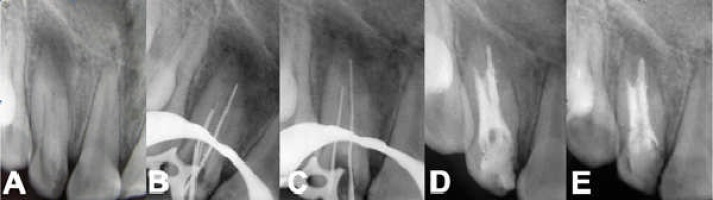
A) Preoperative radiograph of maxillary lateral incisor showing dens invaginatus with periapical radiolucency, B and C) K-files in both canals, D) Radiograph immediately after obturation of primary root canal, and E) one-year follow-up radiograph of maxillary lateral incisor. Radiograph reveals a reduction in size of the radiolucent area

Radiographic examination revealed an apical radiolucency of approximately 6 mm in diameter and an anomalous internal structure consistent with class III dens invaginatus (Figure 2A). The diagnosis was pulp necrosis with chronic apical periodontitis. The contralateral lateral incisor was also checked for clinical and radiographic sign of the same abnormality, but none was detected.

The treatment plan presented was to perform RCT. After rubber dam isolation and gaining access into the pulp chamber, two distinctly separate areas of pulp tissue were found.

**Figure 3:**
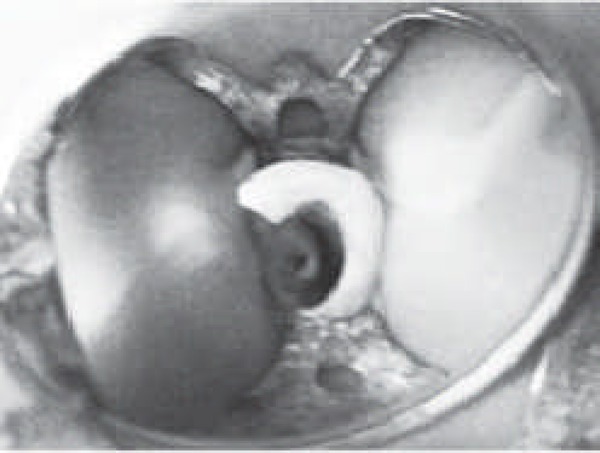
Access opening demonstrating two distinct canal orifices

A central component was surrounded by internal hard tissue; the lateral component appeared to form a c-shaped extending from the mid labial towards the mesial and palatal surface (Figure 3).

After determination of working length (Figure 2 B and C), biomechanical preparation complemented by irrigation with 5.25% sodium hypochlorite, calcium hydroxide paste was applied. The access cavity was temporarily sealed with Cavit.

After one week, patient returned without any symptoms. At this appointment, the tooth was not tender to percussion and the soft tissue in the area was not tender to palpation. After rubber dam placement, the canal was irrigated with 1% sodium hypochlorite and dried with paper point.

The invagination was obturated by lateral condensation of gutta-percha and AH-26 primary root canal was obturated using an injection-moulded thermoplasticized gutta- percha delivery system (Figure 2D).

At one-year follow up, the patient reported no symptoms, the tooth was not tender to percussion and the labial mucosa related to the area was not tender to palpation. The radiography showed reduction in size of the apical radiolucency (Figure 2E). The patient planned for annual recalled as a long term follow up.

## DISCUSSION

Clinicians should be aware of the incidence and methods for treating dens invaginatus. Failure to locate, debride and obturate complex root canal spaces will lead to failure in some cases. According to the classification of Oehlers (1957) the present case was a type 3. However, in this case the invagination did not appear to communicate with the pulp and clinical exploration during root canal treatment corroborated this assumption. Therefore, the etiology of the periapical pathosis in this case was due to the infected primary root canal. However, it is not known how long the root canal had been infected prior to the patient developing symptoms.

Mechanical debridement of the primary root canal was difficult, but the combination of chemo- mechanical instrumentation and the use of calcium hydroxide were sufficient without resorting to surgery. As calcium hydroxide has been reported to successfully eliminate bacteria (20) and stimulate hard tissue repair (21), it was decided to treat the primary root canal with this medicament before obturating the root canal with gutta-percha. The use of a warm gutta-percha technique helped to obturate the root canal system, as it was possible to compact the softened material into the major irregularities within the root canal system (22).

## CONCLUSION

Despite complex anatomy and diagnosis of dens invaginatus, non-surgical root canal treatment was performed successfully.
